# Meningioangiomatosis: A Case Report and Literature Review Emphasizing Diverse Appearance on Different Imaging Modalities

**DOI:** 10.1155/2011/361203

**Published:** 2011-10-09

**Authors:** Osama N. Kashlan, David V. LaBorde, LaKesha Davison, Amit M. Saindane, Daniel Brat, Patricia A. Hudgins, Robert E. Gross

**Affiliations:** ^1^Department of Neurosurgery, Emory University School of Medicine, Atlanta, GA 30322, USA; ^2^Department of Radiology, Emory University School of Medicine, Atlanta, GA 30322, USA; ^3^Department of Pathology and Laboratory Medicine, Emory University School of Medicine, Atlanta, GA 30322, USA

## Abstract

*Purpose*. Meningioangiomatosis (MA) is a rare, benign lesion that commonly mimics other intracranial malformations in clinical presentation and appearance on imaging. The case presented and the literature review performed highlight the importance of combining MRI and CT results to better characterize intracranial lesions and including MA on the list of differential diagnoses of patients presenting with seizures. *Methods*. The case described is of a 19-year-old male with a 10-year history of worsening seizures refractory to multiple drug regimens. MRI revealed an atypical vascular malformation. The patient underwent surgical resection of the epileptogenic cortex. *Results*. Although the radiologic impression of the lesion was a vascular malformation, pathological examination revealed MA. A literature search performed highlights the variability of the appearance of MA on CT and MRI and suggests the utility of the T2 GRE sequence in illustrating the presence of calcification and, in a lesion with other characteristic features, the diagnosis of MA. *Conclusion*. MA can be a difficult diagnosis to make based on imaging findings alone. However, in a patient with a characteristic history and presentation, the presence of a calcified mass on CT and MRI brain susceptibility artifact on a T2 GRE sequence may suggest MA.

## 1. Introduction

Headaches and seizures are the most common clinical presentation in sporadic meningioangiomatosis (MA) [1], and seizures are the sole or predominant clinical problem in 81% of patients with MA [2]. Seizures are refractory to medical therapy in 85% of patients with MA without neurofibromatosis (NF) [3, 4]. Interestingly, the cases associated with NF are often found incidentally and are not associated with seizures [4].

Here, we present a case of MA, emphasizing the difficulty in establishing the diagnosis, the surgical technique used to resect the epileptogenic foci, and the postoperative outcome in terms of minimized frequency of seizures. The case reported demonstrates that MA can mimic several other pathologies on imaging and clinical presentation. It is therefore, imperative to educate clinicians on the possibility of this diagnosis especially in the setting of seizures and nonspecific neuroimaging findings. 

A review of the published literature on MA was done in May 2010 using PubMed to find all articles on meningioangiomatosis, meningeal angiomatosis, meningio-angiomatosis, meningoangiomatosis, or meningo-angiomatosis. In order to be included, case reports and series of patients published in the literature had to be in English and had to include data about the imaging appearance of the lesions in the patients reported either within text and/or figures. The search resulted in 91 papers, 61 which were used in data collection [1–61]. Twenty of the other papers either were not relevant to MA or did not discuss features of the lesion on imaging. The rest of the documents dealt with MA in animals.

## 2. Case Report

### 2.1. History, Presentation, and Examination

The patient was a 19-year-old male with a ten-year history of epilepsy, as well as transient episodes of severe morning headaches with nausea, vomiting, and dizziness. At age nine during a workup for severe headaches, he was found to have a presumed right insular vascular abnormality on imaging. A month later, the patient began having seizures. He was managed medically for ten years; however, during this time, his seizures progressed in frequency from occurring approximately once every six to nine months to almost daily simple or complex partial seizures and rare generalized tonic-clonic seizures despite maintenance on high-dose antiepileptic therapy. Given his refractory and progressive disease, he eventually presented to clinic for consultation about possible surgical management. The only finding on physical examination was distal left upper extremity weakness and hand muscle atrophy resulting from an injury in a motor vehicle accident sustained at the time of a seizure. 

#### 2.1.1. Diagnostics

**Figure 1:**
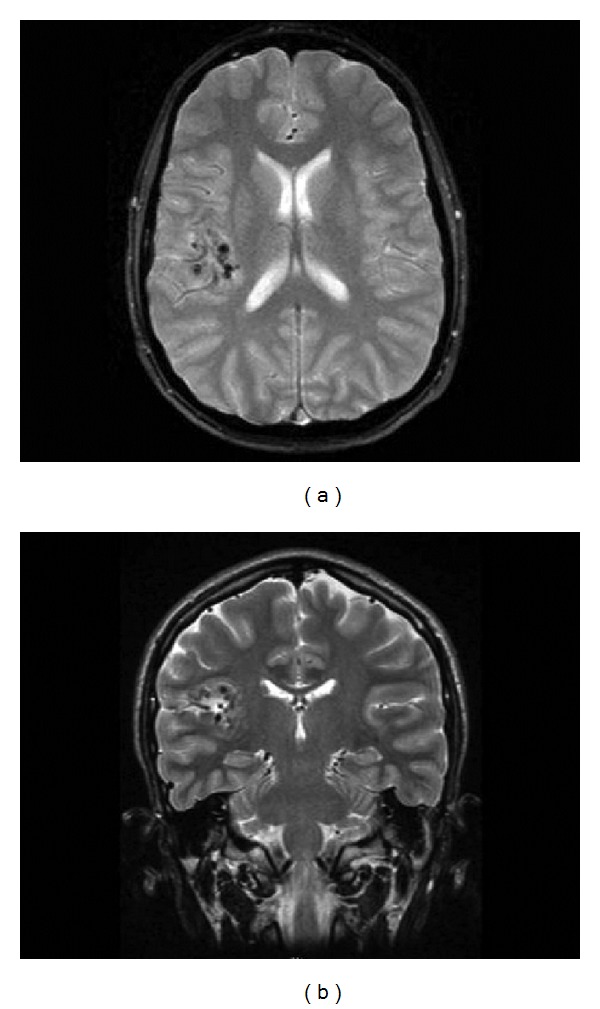
T2-weighted axial (a) and coronal (b) MRI performed 1 year prior to surgery show right posterior frontal flow void with adjacent cortical foci of susceptibility artifact suggesting vascular malformation.

**Figure 2:**
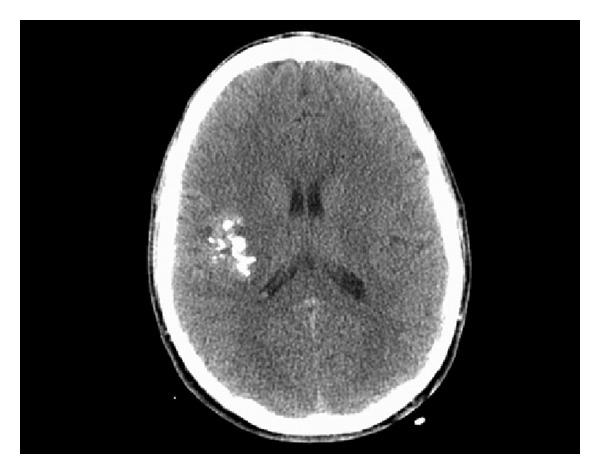
Axial CT without contrast demonstrates right posterosuperior temporal lobe ill-defined clumped calcification.

**Figure 3:**
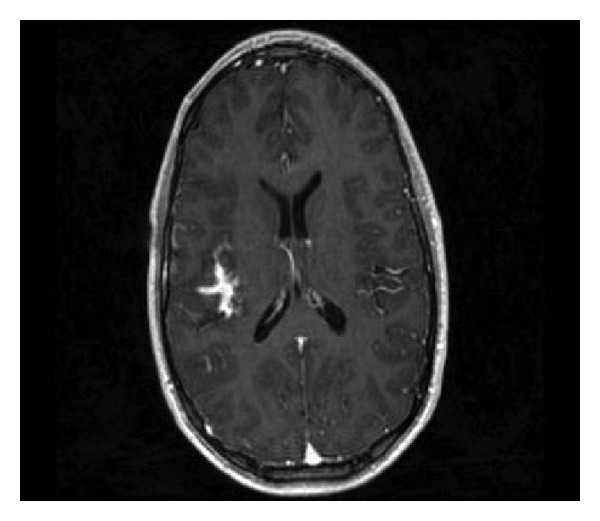
Relatively T1-weighted gradient echo axial MRI with gadolinium contrast shows serpentine enhancement in depths of right sylvian fissure compatible with presumed vascular malformation.

A magnetic resonance imaging (MRI) scan performed one year prior to surgery showed a suspected single right posterior frontal flow void with adjacent cortical foci of susceptibility artifact compatible with calcification or hemosiderin from remote, small hemorrhages suggesting an atypical vascular lesion (Figures 1(a) and 1(b)). The flow void itself had the appearance of a developmental venous anomaly (DVA), but the pattern of adjacent hemosiderin deposition was noted to be unusual for DVA or an associated cavernous malformation. There were no imaging features specific for parenchymal arterial venous malformation (AVM) or dural AV fistula. The vascular abnormality identified in the right frontal operculum was not demonstrated on MRA images. Imaging was repeated and showed the same lesion again felt to likely represent an atypical vascular malformation (Figures 2 and 3). 

#### 2.1.2. Management

The patient was elected to undergo intracranial seizure monitoring with subdural grid and depth electrode placement in order to determine the location of all epileptogenic foci.

**Subdural Grid and Depth Electrode Placement**

A craniotomy was performed, and a 64-contact grid was placed over the lateral surface of his right frontal, temporal, and parietal lobes. Three depth electrodes consisting of 12-contact leads were also placed stereotactically into the amygdala, the anterior/middle hippocampus, and the middle/posterior hippocampus.

**Inpatient Intracranial Seizure Monitoring**

Over the next ten days, the patient remained on the inpatient ward for intracranial seizure monitoring. The patient had 4 seizures that all localized to the area of the abnormality on his MRI scan, providing good evidence for a possible surgical cure with resection.

**Definitive Resection**

The patient was taken back to the operating room for resection. Intraoperatively, the points on the grid with the highest activity were identified. Utilizing navigation, the area was confirmed to localize to the lesion on the MRI scan. The pia was then opened to demarcate the borders of the proposed resection. Subpial dissection was then carried out, and, after initial removal of the superficial cortex, there was a significant amount of fibrous tissue identified. A portion of the tissue was sent for frozen section which the pathologists felt was consistent with MA. A subtotal resection was completed without any apparent complications.

#### 2.1.3. Postoperative Course

**Figure 4:**
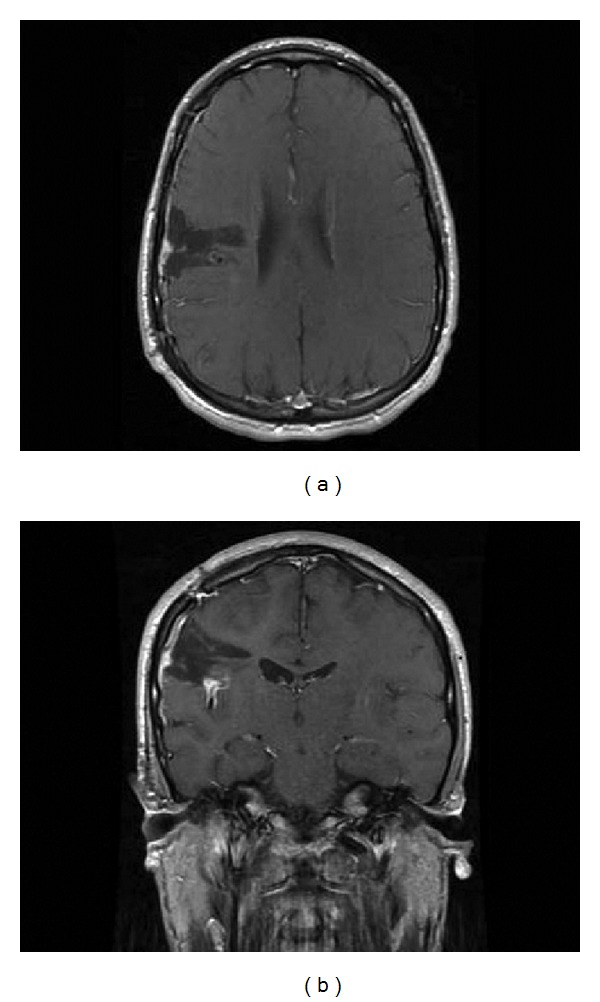
T1-weighted axial (a) and coronal (b) MRI at the 1-year postoperative visit show encephalomalacia, no increase in size of residual lesion, and persistent enhancement in sylvian cistern.

Postoperatively, the patient had some left-sided weakness, dysarthria, and left facial weakness. At one month postoperatively, he was almost completely back to his baseline with only minimal residual left facial weakness. The patient did not experience further complex partial seizures. In the immediate postoperative period, he had occasional auras every two to three days, but at one month postoperatively, this had decreased to once every four to six days. At his 20 month visit, he had no auras and his previously almost daily seizures had been eliminated. He was continued on his antiepileptic medications; however, a few days prior to his one year postoperative visit, he stopped taking his seizure medications for two days and suffered a generalized tonic-clonic seizure. A surveillance MRI performed at this visit showed encephalomalacia and no change in the size of the residual lesion (Figures 4(a) and 4(b)). The patient's seizure medications were resumed, and he has not had any seizures or auras since on dual antiepileptic therapy.

## 3. Pathology

**Figure 5:**
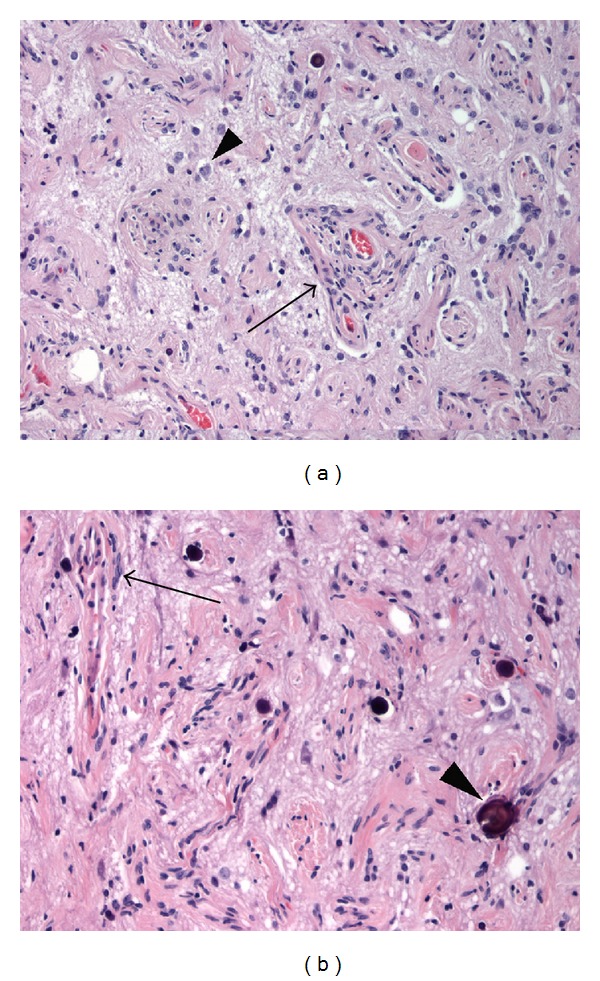
Histologic sections (200x) stained with hematoxylin and eosin (H&E) demonstrate greatly thickened vascular walls (a, arrow) within the cerebral cortex that are concentrically surrounded by meningioma cells, typical of meningioangiomatosis. Pyramidal cells (a, arrowhead) of the architecturally distorted cortex are noted between thickened vascular structures. In other regions (b, arrow), vessels showed fewer surrounding meningioma cells, but greater degrees of collagen, indicating substantial chronicity. Psammoma bodies (b, arrowhead) were scattered throughout.

On pathological examination, there were numerous thickened blood vessels surrounded by sheaths of well-differentiated meningothelial tumor cells, generally ranging from one to five cell layers thick, noted throughout the distorted cerebral cortex (Figures 5(a) and 5(b)). There was extensive fibrosis in the perivascular compartment as well, with bundles of collagen laid down in a concentric laminar pattern. Fibrosis was also noted to extend into the cortical tissue, replacing and displacing brain parenchyma. In between vascular structures, small pieces of intervening cerebral cortex with architecturally and cytologically distorted neurons and abundant reactive gliosis were evident. In some areas, psammoma bodies were numerous. The MIB-1 proliferative rate was exceptionally low, estimated at less than 1%, and a progesterone immunostain was negative. These changes and immunohistochemical results are typical of meningioangiomatosis.

## 4. Discussion

Meningioangiomatosis (MA) is a rare, benign lesion usually affecting the leptomeninges and underlying cerebral cortex but has also been described in the brainstem and thalamus [1, 3, 5]. The pathogenesis of MA is unknown, although several hypotheses have been advanced. It has been proposed that MA is an uncharacterized vascular malformation, a direct invasion of a leptomeningeal meningioma into the brain, or represents a hamartomatous lesion of the leptomeninges and cerebral cortex [3, 6, 62]. Recent reports have implicated specific genetic alterations in the region for the NF type 2 gene [7, 8]. Moreover, multiple case reports demonstrate association of MA with other lesions including meningiomas [9–16], hemorrhage [3, 12, 17, 18], oligodendroglioma [19], encephalomalacia [20], and AVM/meningioma [62].

The patient in this case report presented in a way that is typical for MA without NF. The majority of those affected are children and young adults [3, 21]. MA occurs more frequently in males than in females. [2, 3]. Headaches and seizures are both typical findings for MA [1, 3]. Seizures associated with MA are refractory to antiepileptic drugs in the majority of patients [3]. The most common region affected by MA is the frontotemporal-parietal area [3]; structural lesions in this area are known to lower the seizure threshold [22]. There is a statistically significant predominance of right hemispheric lesions [2, 3]. 

Because of its rarity, information about the characteristics of MA and its appearance on imaging comes mostly from case reports with few numbers of patients. Moreover, to our knowledge, there has not been an attempt to combine data from the different case reports available to determine with better accuracy how these lesions appear on MRI or CT. The literature search performed found 61 reports describing 101 cases of MA that included either a discussion or illustration of the characteristics of the lesion on imaging. The sex, age, side of involvement, lobe involved, and presenting complaint of patients found in literature review are shown in Table 1. From Table 1, it is shown that MA occurs more commonly in males when compared to females, children and young adults when compared to older adults, and on the right side when compared to the left. Moreover, Table 1 shows that seizures predominantly are the main presenting symptom for patients with MA. 

The first imaging modality analyzed was computed tomography (CT). Out of the 101 patients included in the literature search, 68 patients' workups included CT scanning, with 35 patients obtaining contrasted studies. Nine patients had completely normal CT scans. The 59 remaining patients were evaluated for density of the lesions on CT relative to the surrounding brain parenchyma, enhancement, and presence of calcification. The results are shown in Table 2 and illustrate how difficult it is to diagnose MA using CT alone. However, some trends can be elicited from the data: over half of the lesions appear hypodense on CT and enhance with contrast administration. However, the strongest indicator for the presence of MA on CT was calcification within mass with almost 90% of lesions exhibiting at least some degree of calcification.

The second imaging modality examined was magnetic resonance imaging (MRI). From the 101 patients included in the literature search, 79 patients' workups included MR imaging. Two patients had completely normal MRI scans. The 77 remaining patients were evaluated for intensity of lesions on T1- and T2-weighted sequences, enhancement on T1-weighted sequences, presence of susceptibility artifact, and surrounding edema. The results are summarized in Table 3. As shown in Table 3, MA can have multiple appearances on MRI. MA was shown to be most commonly hypointense on T1, hyperintense on T2, with about 80% of lesions demonstrating at least some level of enhancement on T1. MA was also associated with presence of susceptibility artifact and edema in about half of the cases.

The third imaging modality analyzed was cerebral angiography. The results are summarized in Table 4. As shown in Table 4, most cerebral angiograms performed on patients with MA are normal, but rarely hypervascularity, an abnormal vessel or an avascular mass can be seen.

MA can be even more diverse in its presentation on imaging because of the different morphologies that lesion can assume; for example, MA can be gyriform [2, 3, 23–30], or can be associated with a cystic component [3, 11, 13, 19, 25, 31–33]. Some studies proposed certain properties that may help clinician in diagnosing lesion via imaging. Yao et al. concluded that gyriform hyperintensity on a FLAIR sequence is the main MRI feature of MA [30]. Rokes et al. suggested the possibility of using magnetic resonance spectroscopy and fluorodeoxyglucose positron emission tomography in helping with the diagnosis of MA [34]. 

Based on the findings of the literature search conducted herein, the most common findings in MA are enhancement on T1-weighted MR imaging, and calcification on CT, with a prevalence of 79.6%, and 89.6%, respectively. With the exception of these characteristics, no other generalizations can be made. The reported findings are observation based and as such are subject to bias and intra-observer variability given that the studies were interpreted by different radiologists. In addition, some reports identified had inadequate figures or descriptions and were limited as only select or representative images are shown as opposed to the entire study. Moreover, missing or “unspecified” data could skew the results particularly if the omitted images are not randomly distributed, and the majority of them were to demonstrate a particular phenomenon. For example, if the 27 “unspecified” study findings in reality demonstrated edema, this would change the results drastically. 

However, with the acknowledgement of these limitations, the literature review conducted herein does identify certain trends that could be important in helping diagnose MA in the future. Several of the cases where a T2 GRE MRI sequence was obtained identified susceptibility artifact which correlated well with CT findings of calcification. That is, the MRI finding of susceptibility artifact appears to correlate with the presence of calcification on the CT. While chronic hemosiderin from cavernous malformations will show susceptibility artifact on T2-GRE, it will not typically show calcification on CT. Cavernous malformations will also usually be a single focus rather than a gyriform morphology and will typically have a “popcorn” appearance of T2 hyperintensity and hypointensity on standard T2-weighed imaging. The T2 GRE sequence has become a fairly routine part of brain MRI protocols and is particularly important for epilepsy protocols. This case report and the review of the literature suggest yet another use for the T2 GRE sequence, suggesting the presence of calcification, and in a lesion with other characteristic features, the diagnosis of MA.

Another imaging characteristic of MA identified in the literature review is that a significant number of the lesions exhibit at least some edema and mass effect. This is in contrary to some reports that describe that MA is typically a mass with relatively little mass effect and edema for its size due to its origin as a hamartomatous or vascular process rather than a malignant neoplasm. In our search, almost 46% of cases showed at least some edema in the area surrounding lesion; therefore, clinicians should not rule out MA simply by the presence of edema or mass effect.

CT and MRI are both important modalities for establishing MA as a potential diagnosis. In our case, both calcification on the head CT and T2 GRE susceptibility artifact on the MRI of the brain were present and in retrospect perhaps should have moved up MA in our differential. Moreover, the present case illustrates well that what may appear to be flow voids on MRI preoperatively may simply be signal void from coarse calcification, a consideration that should be kept in mind. 

## 5. Conclusion

MA can be a difficult diagnosis to make based on imaging findings alone and can be mistaken on MRI of the brain for other pathologies such as meningiomas, cavernous malformations, and other vascular abnormalities. A diagnosis of MA should be considered when a young patient presents with a headache, seizure, or with increasing difficulty in controlling a known seizure disorder in the setting of a calcified mass on CT and difficult to characterize mass on MRI. If calcification is present on a CT scan of the head, the possibility of a diagnosis of MA should be considered, especially if the mass is noted to be consistent with an atypical vascular malformation on MRI of the brain, as was the situation in this case report. The case reported herein and the review of the literature suggest that the presence of MRI brain susceptibility artifact on a T2 GRE sequence in a patient with a characteristic history and presentation may suggest the presence of a calcified lesion and possibly MA if seen in conjunction with the other typical findings on imaging; thus, in this patient population, when T2 GRE susceptibility artifact is present, this should prompt evaluation with a CT scan of the head if one has not already been obtained. 

## Figures and Tables

**Table 1:** Patient characteristics. The below table summarizes the aggregate characteristics of patients appearing in reports that met inclusion criteria for the review of the literature. Total patients in literature review is 101.

Patient characteristics^a^	
Sex (*n* = 100)	
Unspecified	1
Male	58 (58%)
Female	42 (42%)
Age (*n* = 100)	
Unspecified	1
Less than or equal to 2 years of age	5 (5%)
Between 2 to 10 years of age	32 (32%)
Between 11 to 20 years of age	35 (35%)
Between 21 to 30 years of age	13 (13%)
Between 31 to 40 years of age	9 (9%)
Between 41 to 50 years of age	2 (2%)
Between 51 to 60 years of age	3 (3%)
Side of involvement (*n* = 93)	
Unspecified	6
Multiple lesions affecting both sides	2
Right	49 (52.7%)
Left	41 (44.1%)
Bilateral/midline involvement	3 (3.2%)
Lobe involved (*n* = 84)	
Unspecified	3
Multilobe involvement by single lesion (frontotemporal, temporoparietal, etc.)	14
Frontal	33 (40.2%)
Temporal	33 (40.2%)
Occipital	5 (6.1%)
Parietal	9 (11.0%)
Brainstem	1 (1.2%)
Cerebellar vermis	1 (1.2%)
Presentation (*n* = 101)	
Seizure/epilepsy	75 (74.3%)
Seizure/epilepsy plus another complaint	83 (82.3%)

^a^Those reports that lack particular data or details are denoted as “unspecified” and are subtracted from the total number of patients (the denominator) when calculating percentages. There were two case reports that contained multiple MA lesions that affected both right and left hemispheres and, therefore, were not included in calculating percentages for side of involvement. Number of patients included in calculating percentages in each subset is denoted by (*n*).

**Table 2:** Meningioangiomatosis appearance on computed tomography. Summary of the CT head imaging findings of meningoangiomatosis cases reported in the literature.

Computed tomography^b^	
Total patients in literature search: 101. Patients with CT: 68. Normal CT: 9. # of patients left: 59	
Density (*n* = 41)	
Unspecified	18
Hyperdense	15 (36.5%)
Hypodense	22 (53.7%)
Mixed	4 (9.8%)
Enhancement (*n* = 35)	
Unspecified	24
Yes	21 (60%)
No	14 (40%)
Calcification (*n* = 48)	
Unspecified	11
Yes	43 (89.6%)
No	5 (10.4%)

^b^Those reports that lack particular data or details are denoted as “unspecified” and are subtracted from the total number of patients (the denominator) when calculating percentages. Number of patients included in calculating percentages in each subset is denoted by (*n*).

**Table 3:** Meningioangiomatosis appearance on magnetic resonance imaging. Summary of the MR head imaging findings of meningoangiomatosis cases reported in the literature.

Magnetic resonance imaging^c^	
Total patients in literature search: 101. Patients with MR: 79. Normal MR: 2. # of patients left: 77	
T1 intensity (*n* = 46)	
Unspecified	31
Hyperintense	2 (4.3%)
Isointense	9 (19.6%)
Hypointense	26 (56.5%)
Mixed	9 (19.6%)
T2 intensity (*n* = 58)	
Unspecified	19
Hyperintense	29 (50.0%)
Isointense	3 (5.2%)
Hypointense	8 (13.8%)
Mixed	18 (31.0%)
T1 enhancement (*n* = 49)	
Unspecified	28
Yes	39 (79.6%)
No	10 (20.4%)
Artifact (*n* = 46)	
Unspecified	31
Yes	26 (56.5%)
No	20 (43.5%)
Edema (*n* = 50)	
Unspecified	27
Yes	23 (46%)
No	27 (54%)

^c^There are multiple reports which do not signify certain details. These cases are denoted as “unspecified” and are subtracted from the total number of patients when calculating percentages. Number of patients included in calculating percentages in each subset is denoted by (*n*).

**Table 4:** Characteristics of meningioangiomatosis on cerebral angiography.

Cerebral angiogram (*n* = 30)	
Normal	22 (73.3%)
Hypervascularity	1 (3.3%)
Abnormal vessel	2 (6.7%)
Avascular mass	5 (16.6%)

## Abbreviations

AVM: Arteriovenous malformation
CT: Computed tomography
DVA: Developmental venous anomaly
EEG: Electroencephalography
MA: Meningioangiomatosis
MIB1: Mindbomb homolog 1
MRI: Magnetic resonance imaging
NF: Neurofibromatosis.
